# Small mammal responses to fire severity mediated by vegetation characteristics and species traits

**DOI:** 10.1002/ece3.8918

**Published:** 2022-05-19

**Authors:** Kathryn Culhane, Rahel Sollmann, Angela M. White, Gina L. Tarbill, Scott D. Cooper, Hillary S. Young

**Affiliations:** ^1^ Department of Ecology, Evolution, and Marine Biology University of California Santa Barbara California USA; ^2^ Department of Wildlife, Fish, and Conservation Biology University of California Davis California USA; ^3^ Department of Ecological Dynamics Leibniz Institute for Zoo and Wildlife Research Berlin Germany; ^4^ Pacific Southwest Research Station USDA Forest Service Davis California USA

**Keywords:** community structure, fire severity, functional trait, resource use, small mammal

## Abstract

The frequency of large, high‐severity “mega‐fires” has increased in recent decades, with numerous consequences for forest ecosystems. In particular, small mammal communities are vulnerable to post‐fire shifts in resource availability and play critical roles in forest ecosystems. Inconsistencies in previous observations of small mammal community responses to fire severity underscore the importance of examining mechanisms regulating the effects of fire severity on post‐fire recovery of small mammal communities. We compared small mammal abundance, diversity, and community structure among habitats that burned at different severities, and used vegetation characteristics and small mammal functional traits to predict community responses to fire severity three years after one mega‐fire in the Sierra Nevada, California. Using a model‐based fourth‐corner analysis, we examined how interactions between vegetation variables and small mammal traits associated with their resource use were associated with post‐fire small mammal community structure among fire severity categories. Small mammal abundance was similar across fire severity categories, but diversity decreased and community structure shifted as fire severity increased. Differences in small mammal communities were large only between unburned and high‐severity sites. Three highly correlated fire‐dependent vegetation variables affected by fire and the volume of soft coarse woody debris were associated with small mammal community structures. Furthermore, we found that interactions between vegetation variables and three small mammal traits (feeding guild, primary foraging mode, and primary nesting habit) predicted community structure across fire severity categories. We concluded that resource use was important in regulating small mammal recovery after the fire because vegetation provided required resources to small mammals as determined by their functional traits. Given the mechanistic nature of our analyses, these results may be applicable to other fire‐prone forest systems, although it will be important to conduct studies across large biogeographic regions and over long post‐fire time periods to assess generality.

## Graphical abstract

Small mammal community composition varies across fire severities; this variation can be explained by a combination of functional traits and vegetation characteristics.

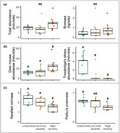

## 1 INTRODUCTION

The world is burning at an alarming rate. Across western North America, wildfires have become larger and more frequent over the past three decades (Abatzoglou & Williams, 2016; Schoennagel et al., 2017; Stephens et al., 2014; Yue et al., 2013). In California alone, the 2020 wildfire season accounted for five of the largest wildfires on record, often termed “mega‐fires” owing to their severity and extent. Yet, despite the strong potential for these shifts in fire regimes to affect vertebrate communities, we have limited information on how vertebrate wildlife is affected by changes in forest fire size and severity, much less the mechanisms that drive these effects and how they may vary across functional groups (Jones & Tingley, 2021). In particular, determining the effects of fire severity on forest vertebrates is critical both because of the needs for their conservation and for the many roles they play in regulating plant communities, forest regeneration, trophic structure, and other ecosystem functions (Furnas et al., 2021; Morrison et al., 2012; Volkmann et al., 2020). Specifically, studies on high‐severity fire effects on mammals are needed: a recent meta‐analysis on fire‐prone forests of the US found only two studies of high‐severity fire impacts on small mammals, despite the roles that small mammals play in forest ecosystems (Fontaine & Kennedy, 2012).

Small mammals are critical for the functioning of forest ecosystems, including mixed conifer forests. Small mammals can modify the structure of forest vegetation through seed predation and dispersal (Briggs et al., 2009; Vander Wall, 2008), and are key agents for the dispersal of ectomycorrhizal fungi (Pyare & Longland, 2001). In addition, small mammals constitute food for predators, including rare North American species such as the Spotted Owl (*Strix occidentalis*) and Pacific fisher (*Pekania pennanti*) (Carey et al., 1992; Zielinski & Duncan, 2004), and serve as vectors or hosts for multiple pathogens (Ostfeld et al., 2018; Stephens et al., 2016). Given the roles that small mammals play in forests, it is important to delineate the effects of fire severity on their community structure and function (Kirkman et al., 2013).

In general, small mammal community structure shifts after a wildfire, although the observed patterns often have been inconsistent. Fire can decrease small mammal diversity by favoring generalist species over specialists (Zwolak & Foresman, 2007), but post‐fire decreases in diversity are not always observed (Roberts et al., 2015), and observed post‐fire abundance patterns are highly variable across systems (Borchert et al., 2014; Converse, Block, et al., 2006; Fisher & Wilkinson, 2005; Hutchen et al., 2017).

Fires alter forest vegetation according to fire severity, thereby changing the availability of vegetation resources for small mammals. As a measure of organic material loss, increasing fire severity reflects greater vegetation mortality. Over longer timescales, fire severity also shapes vegetation structure by regulating light competition, soil nutrients, growth of ruderal species, and accumulation of dead vegetative matter (Webster & Halpern, 2010). High‐severity fire often engenders stronger post‐fire increases in shrub and herbaceous vegetation cover than lower severity fire (Webster & Halpern, 2010), and can influence leaf litter and coarse woody debris inputs (Apigian et al., 2006).

In turn, these vegetation shifts influence small mammals by regulating the availability of key resources. Vegetation, downed wood, and litter cover all provide key resources to small mammals, in the form of protection from predators (Powell & Banks, 2004; Torre & Díaz, 2004), nesting sites (Innes et al., 2007; McComb, 2003), and high‐quality foraging habitat (Bos & Carthew, 2003; Jia‐bing et al., 2005; Reid, 2006; Whitaker, 1996).

Some studies have reported differences in small mammal responses between moderate‐ and high‐severity fire in conifer forests (Kyle & Block, 2000; Roberts et al., 2008), whereas others show negligible differences (Borchert et al., 2014). Further, the effects of fire on small mammals as mediated through vegetation changes appear to vary across mammal species (Converse, White, et al., 2006; Fontaine & Kennedy, 2012; Kalies et al., 2010; Zwolak, 2009). For example, meta‐analyses of small mammal responses to fire in North America show that the deer mouse (*Peromyscus maniculatus*) and white‐footed mouse (*P*. *leucopus*) generally increase in abundance after fire, whereas the southern red‐backed vole (*Myodes gapperi*) decreases in abundance after fire (Fontaine & Kennedy, 2012; Zwolak, 2009).

Globally, interspecific variation in small mammal responses to fire may be explained by small mammal functional traits, especially those corresponding to resource use. Several small mammal traits are hypothesized to correspond with post‐fire shifts in community structure and thereby explain species‐specific patterns (Ceradini & Chalfoun, 2017; Kelly et al., 2010; Plavsic, 2014). In particular, traits such as diet, foraging mode, locomotion, and nesting habit are related to vegetation resource use and therefore likely to respond to shifts in vegetation after fire (Flynn et al., 2009). Other traits such as reproductive rate, home range size, and longevity also have been linked to immediate post‐fire responses because they directly influence survival and recolonization ability (Whelan et al., 2002). Body size has also been correlated with post‐fire survival (Griffiths & Brook, 2014), probably because it is related to life‐history traits directly associated with colonization, reproductive output, and survival (Hutchings et al., 2012).

The objective of this study was to clarify how relationships among fire severity, vegetation characteristics, and small mammal traits might shape post‐fire small mammal community structures. We used a model‐based fourth‐corner framework to examine these relationships, given that trait‐based approaches that incorporate key habitat variables are particularly well‐suited for revealing the mechanistic underpinnings of post‐fire recovery (Driscoll et al., 2010; McGill et al., 2006). We compared nine sites within each of three fire severity categories (unburned, low‐moderate, high) because robust spatial replication is crucial for mechanistic studies to account for habitat variation (Griffiths & Brook, 2014).

**FIGURE 1:**
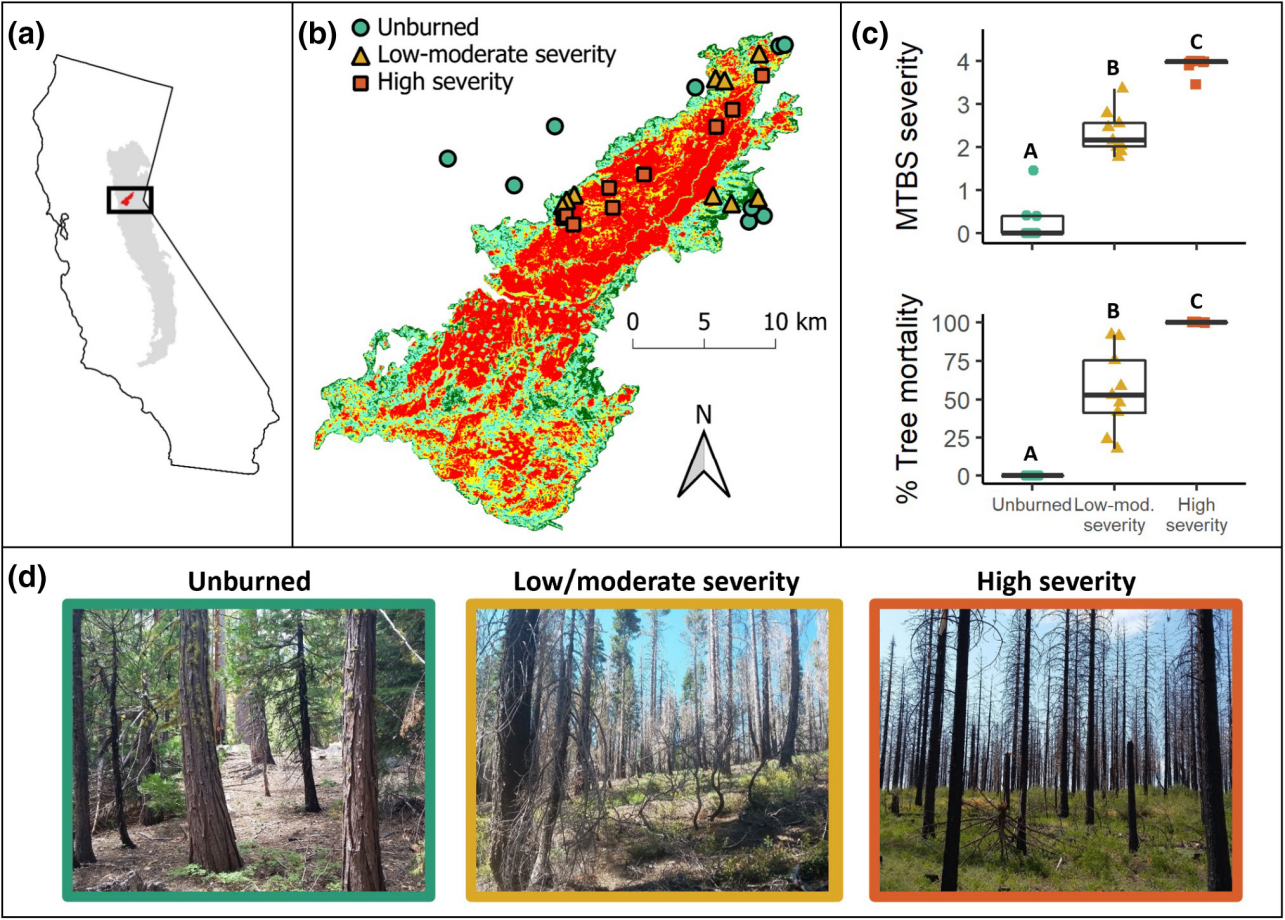
Sites across a fire severity gradient. (a) The area of the 2014 King Fire is shown in red, and the Sierra Nevada ecoregion within California is shown in light gray. (b) Sites were categorized by three fire severity categories (unburned, low/moderate severity, and high severity). (c) Monitoring Trends in Burn Severity scores and in situ % tree mortality was different among categories. Box plots show the median and upper/lower quartiles. Fire severity categories with the same letter are not significantly different. (d) Sites shifted from a mixed yellow‐pine forest to a shrub‐dominated understory across the severity categories

Specifically, we examined the possible drivers of small mammal community structure after the King Fire, a mega‐fire in the north‐central Sierra Nevada of California. The King Fire burned 39,545 ha in September and October 2014, during a historic California drought (Figure 1a). Over 50% of the King Fire area burned at high severity, including one continuous 13,683‐ha high‐severity patch. The extent of high‐severity fires in the north‐central Sierra Nevada of California has increased over the past three decades, in part due to timber harvesting practices and past fire suppression (Agee, 1998; Miller et al., 2009). More recently, fire regime shifts are being exacerbated by climate change through a lengthened fire season, warming temperatures, and increased drought frequency (Westerling et al., 2006). As one of the first well‐publicized mega‐fires in California, the King Fire was a seemingly anomalous event that is quickly becoming the norm.

We compared small mammal communities in areas that did not burn versus areas that burned at high and low/moderate severities three years after the King Fire. Specifically, we examined differences among fire severity categories in relation to the following questions: (1) How did small mammal abundance, diversity, and community structure respond to fires of low/moderate versus high severity? (2) Which vegetation characteristics were associated with fire‐related shifts in mammal community structure? (3) Which small mammal traits explained variation in mammalian responses to fire severity?

We expected that the effects of fire severity on small mammal community structure would be mediated by resource use, as shown by relationships between vegetation characteristics and small mammal traits. In particular, we expected to see post‐fire increases in small mammal abundance, decreases in diversity, and shifts in community structure, consistent with previously reported patterns in North America (Zwolak & Foresman, 2007) and Australia (Griffiths & Brook, 2014), with stronger mammalian responses in high‐severity than in low/moderate‐severity habitat (Fontaine & Kennedy, 2012; Pastro et al., 2014). Based on previous studies in North America and elsewhere, we hypothesized that vegetation shifts in shrub cover (Borchert et al., 2014; Torre & Díaz, 2004), forb and grass cover (Plavsic, 2014; Powell & Banks, 2004), litter cover (Bos & Carthew, 2003; Greenberg et al., 2007), density of live trees (Lobo, 2014; Miklós & Îiak, 2002), and amount of well‐decayed coarse woody debris (Fauteux et al., 2012; Jia‐bing et al., 2005; McComb, 2003) would be associated with fire severity and thereby predict post‐fire small mammal community structure. We also hypothesized that feeding guild, foraging mode, and nesting habit would explain vegetation‐related variation in post‐fire small mammal community structure, due to the traits’ associations with the use of vegetation resources (Flynn et al., 2009; Griffiths & Brook, 2014; Plavsic, 2014).

## 2 METHODS

### 2.1 Study location

This study was conducted in Eldorado National Forest (38°45′N; 120°20′W), near Placerville, California, within the footprint of the King Fire, which burned in the fall of 2014 (Figure 1). Within the King Fire perimeter, fire severity ranged from low to high (Figure 1b) with vegetation in high‐severity areas shifting drastically from a mixed conifer forest with limited understory before the fire to a homogenous shrub‐dominated understory with skeletal trunks after the fire (Figure 1d). Sampled sites ranged in elevation from 1300–1900 m, and pre‐burn vegetation consisted of lower montane mixed conifer forest. Mixed conifer forests are characterized by a Mediterranean climate with wet winters and dry summers. Common tree species at the study sites included yellow pine (*Pinus ponderosa* and *P*. *jeffreyi*), sugar pine (*P*. *lambertiana*), white fir (*Abies concolor*), incense cedar (*Calocedrus decurrens*), Douglas‐fir (*Pseudotsuga menziesii*), black oak (*Quercus kelloggii*), and red fir (*A*. *magnifica*). The most common post‐fire species were chaparral shrubs such as deer brush (*Ceanothus integerrimus*), mountain whitethorn (*C*. *cordulatus*), greenleaf manzanita (*Arctostaphylos patula*), and prostrate ceanothus (*C*. *prostratus*).

### 2.2 Study sites and experimental design

In summer 2017 we established 27 sampling sites across three fire severity categories, with nine unburned sites (located outside the fire boundary), nine low/moderate‐severity sites, and nine high‐severity sites (Figure 1b). Sites were selected using elevation data and remotely sensed vegetation classes from the California Wildlife Habitat Relationships program (CWHR) (Mayer & Laudenslayer, 1988), although, as detailed below, burn categories were subsequently validated using both field data and Landsat‐derived burn severity imagery.

All sites were established in publicly‐owned areas with no recent pre‐fire logging or post‐fire salvage logging and located at least 50 m from the nearest road, stream, or dissimilar habitat type, such as a clear‐cut. According to Fire and Resource Assessment Program (FRAP) fire perimeter data, none of the sites experienced wildfire or controlled burning within the century before the King Fire (Fire & Resource Assessment Program, 2020). Slopes within all sites did not exceed 30 degrees. Sites were located >100 m from each other (except for two adjacent plots separated by a dirt road), and the average distance from each site to the nearest site was 1.4 km. Sampling took place from late June to early September 2017. To minimize seasonal effects associated with sampling throughout the summer, triplicate unburned, low/moderate‐severity, and high‐severity sites were sampled simultaneously (e.g., one site within each burn category sampled at each sampling time). All mammal and vegetation surveys at a single site were conducted within 4–5 days. The climate was consistently hot and dry at all sites (15–40°C), with no precipitation throughout the sampling period.

We established the similarity of vegetation at our sites before the King Fire and compared pre‐fire to post‐fire conditions using spatial data products from the Landscape Fire and Resource Management Planning Tools program (LANDFIRE) developed in 2012 (LF 1.3.0) and 2014 (LF 1.4.0) (Rollins & Frame, 2006). We also validated differences in burn characteristics by vegetation characteristics after the fire. Details of these methods and analysis of the effects of fire are summarized in Figures 1 and S1, and all analyses showing the strong differences in tree cover and mortality, and vegetation types, across fire severity categories, are summarized in Appendix S1. Essentially high‐severity fires are shown to cause major losses in canopy cover and increased tree mortality, whereas low/moderate‐severity fires cause only modest differences.

### 2.3 Vegetation surveys

We used five measures of vegetation to examine relationships between small mammal community structure and environmental conditions: density of live trees (field methods detailed above), litter cover, cover of understory shrubs, cover of understory grasses and forbs, and volume of coarse woody debris (CWD). All vegetation data were taken along the same two 50‐m transects used for estimating tree mortality at each site. The vegetation transects ran parallel to two sides of the small mammal trapping grid and were located 10–20 m away from the grid edge. To characterize litter and understory vegetation cover, we estimated the percent cover of litter material and live vegetation up to 1 m tall within 1‐m^2^ quadrats located every 5 m along each transect (10 quadrats per transect, 10 m^2^ total). All live understory vegetation was categorized as tree, shrub, grass, or forb, with percent cover being estimated separately for each life form.

Coarse woody debris (CWD) also was surveyed along the same vegetation transects, using line‐intercept methods (Waddell, 2002). For each piece of CWD (defined as wood longer than 1 m with a diameter at the point of transect intersection >12.5 cm), we recorded its length, smallest diameter, and largest diameter. The volume of CWD per m^2^ was determined using Smalian's volume formula and DeVries’ formula, which converts line‐intercept data into volume per unit area (DeVries, 1973; Waddell, 2002). We also recorded the decay class for each piece of CWD (ranging from 1 = intact sound wood to 5 = soft powdery wood with no structural integrity, (Maser et al., 1979)). Only well‐decayed CWD in decay classes 3–5 were included in analyses because this material is used more heavily by small mammals than less‐decayed wood (Jia‐bing et al., 2005).

### 2.4 Small mammal sampling

At each site, we sampled small mammal communities within one 90 × 90 m trapping grid, with traps placed 10 m apart (100 traps per grid). Grids were arranged by alternating large (7.5 × 9 × 23 cm) and extra‐large (10 × 11.5 × 38 cm) Sherman traps baited with a mixture of oats, peanut butter, bird seed, and molasses. To improve trap success, we allowed animals to acclimate by pre‐baiting traps and holding them open for three consecutive nights. We then sampled each grid for three consecutive trap nights (maximum of 300 trap nights per site). Traps were opened between 17:00 and 19:00 and closed between 09:00 and 11:00, with no daytime trapping effort due to heat. Captured small mammals were identified as species using external morphological characteristics and marked with unique ear tags, or for shrews only, clipped fur. We also recorded each mammal's mass and age class, and noted any nonfunctional or sprung traps to assess trapping effort. Following Beauvais and Buskirk (1999), we considered nonfunctional traps (traps that no longer operated properly, for example, due to severe disturbance by bears) as having 0 effort and sprung traps (traps that appeared fully functional but were found shut yet empty) as having an effort of 0.5. Although traps were primarily open at night, several diurnal species (chipmunks and ground squirrels) were regularly captured, probably because traps were consistently open for a few hours after sunrise and before sunset at all sites. Because trapping times were standardized across sites, our sampling scheme allowed a comparison of the relative abundances of all captured species across sites.

### 2.5 Small mammal abundance and diversity

From the trapping data, we calculated small mammal abundance as the number of unique individuals captured over each sampling period at each site, representing the minimum number of animals known to be alive (MNKA). To confirm similarity in capture success among burn severities, we compared the recapture rate of marked individuals (number of recaptured individuals per number of total captures) among the three fire severity categories using a Kruskal–Wallis test and found no significant differences. Although a mark‐recapture (M‐R) model would have been preferable, data for most species were simply too sparse to conduct M‐R analysis. To minimize seasonal variation across sites, only adult animals were included in all analyses.

Abundance estimates for all analyses were standardized by trapping effort, so that abundance was measured in individuals per trapping grid per trap night (~300 per grid across all three trap nights, although usually 2–5% lower when nonfunctional and sprung traps were accounted for). We calculated the biomass of each species at each site as the product of the species’ abundance multiplied by the mean body mass from field measurements of adults. Total small mammal abundance and total biomass, and the abundances of individual species, were compared among fire severity categories using Kruskal–Wallis tests followed by Bonferroni‐corrected Dunn's tests.

To characterize the diversity of the small mammal community, we calculated species richness and evenness for each site and compared these across fire severity categories using ANOVA with post hoc pairwise comparisons using Tukey's HSD tests. Rarefied species richness was estimated by individual‐based rarefaction using the rarefy function in the R package vegan (Oksanen et al., 2018; Thompson et al., 2007; Willott, 2001). We also calculated Pielou's index of species evenness (Pielou, 1966).

### 2.6 Small mammal community structure

We used a combination of model‐based and association‐based methods for multivariate analysis of the small mammal community. Specifically, we built a multivariate generalized linear model (GLM) to examine differences in small mammal community structure among fire severity categories and used nonmetric multidimensional scaling (NMDS) ordination for the visualization of patterns across burn categories. GLMs are often used for analyzing multivariate abundance data because they account for strong mean‐variance relationships and strong correlations among response variables (Wang et al., 2012; Warton, Foster, et al., 2015).

We built the first multivariate GLM using the fire severity category as a predictor variable and small mammal species’ abundances as response variables (GLM_severity_). We assumed a negative binomial distribution of abundance data. We included Principal Components of Neighborhood Matrix (PCNM) distances across sampling sites as a metric of spatial autocorrelation in our models (Dray et al., 2006). The model was created with the function manyglm in R package mvabund, using the approach developed by Wang et al. (2019). Multivariate test statistics were calculated using the Score statistic because our data included means of abundances for rare species, and we accounted for correlations between species by shrinking the sample correlation (Warton, 2011). To test model significance, we calculated p‐values using the PIT‐trap bootstrapping method for resampling of rows with the anova.manyglm function (Warton et al., 2017). We also calculated univariate test statistics and *p*‐values to determine which species were driving patterns.

To visually represent differences in community structure among fire severity categories, we conducted NMDS on the abundance of all captured species across sites using the metaMDS function in the R package vegan (Oksanen et al., 2018). Raw abundance values were standardized using the Hellinger method, which standardizes abundance by site and then applies a square root transformation (Legendre & Gallagher, 2001). We then generated a Bray‐Curtis dissimilarity matrix and produced a 3‐dimensional ordination solution. To corroborate the results of GLM_severity_, we evaluated the similarity in community structure among fire severity categories using a permutational multivariate analysis of variance of Hellinger‐standardized abundance (adonis function in vegan (Oksanen et al., 2018)). Post hoc pairwise comparisons of multivariate abundance data for burn categories were conducted using the function pairwise.adonis in R package pairwiseAdonis, followed by Bonferroni corrections (Martinze Arbizu, 2020).

### 2.7 Vegetation drivers

We explored associations between five vegetation variables (shrub cover, forb and grass cover, live tree density, volume of coarse woody debris, and litter cover) on small mammal community structures. To visualize how these variables corresponded to patterns in small mammal community structures, we plotted vectors for each vegetation variable onto the NMDS plot using the function envfit in vegan (Oksanen et al., 2018).

To determine the vegetation characteristics that were associated with fire severity, we compared data for fire severity categories using Kruskal–Wallis tests followed by Bonferroni‐corrected Dunn's tests. We also checked for correlations among the vegetation variables using Spearman's rank correlation coefficients. For variables with coefficients >0.5, we used principal component analysis (PCA) to collapse the variables into a single metric (the first principal component, PC1) that explained a large portion of the variance in vegetation variables, which we incorporated into our model.

To determine which vegetation variables predicted most of the variation in small mammal community structure, we built a second multivariate GLM using PC1 and the remaining vegetation variables as predictors and transformed small mammal species’ abundances as response variables (GLM_veg_). This GLM also included PCNM distances to account for spatial autocorrelation (described above). We used Akaike information criterion (AIC) selection to determine which combination of predictors’ best‐explained variation in the small mammal data, and used the function summary.manyglm with PIT‐trap bootstrapping to test the significance of each predictor in the final model.

### 2.8 Small mammal functional traits

We hypothesized that small mammal functional traits related to resource use would be correlated with fire‐related vegetation changes. Specifically, we explored the following three resource use traits: feeding guild, primary foraging mode, and primary nesting habit (Ceradini & Chalfoun, 2017; Flynn et al., 2009). Although other traits might also be important, they are either correlated with these traits (e.g., body size) or poorly known across all species (e.g., dispersal distance and fecundity). To account for variability in the information provided by different literature sources (Fitzsimmons, 2013), trait information was collated from two field guides and species accounts from the American Society of Mammalogists (Table S1). Feeding guild was characterized as herbivore, omnivore, or insectivore; primary foraging mode was recorded as ground, scansorial, or arboreal; and primary nesting habit was recorded as a tree, hollow (aboveground, e.g., rock crevices or brush piles), or burrow (underground).

To examine relationships between small mammal traits and vegetation variables, we used a model‐based fourth‐corner approach. Within this framework, three matrices representing site‐species abundance data, site‐environmental data, and species trait data are used to calculate a fourth matrix (“fourth corner”) that estimates relationships between environmental and trait variables (Brown et al., 2014). We created our fourth‐corner model (GLM_trait_) using the traitglm function in mvabund, which predicts species abundance using the environment by trait associations (Warton, Shipley, et al., 2015). We assumed a negative binomial distribution of abundance data. Because this framework does not yet allow offsets to account for trapping effort, we used effort‐standardized abundances in our site‐species matrix. We only included vegetation variables that were significant in GLM_veg_. For model selection, we used the LASSO penalty to remove correlation coefficients that did not reduce Bayes Information Criterion (BIC) (Brown et al., 2014). We visualized the model results by creating a heat map of the remaining standardized fourth‐corner coefficient estimates. To test for model significance, we calculated a p‐value using the PIT‐trap bootstrapping method for resampling of rows with the anova.traitglm function (Brown et al., 2014).

All statistical analyses were performed using the program R 3.4.4 (R Core Team, 2018). The maps in Figure 1 were created using QGIS (QGIS Development Team, 2018).

## 3 RESULTS

### 3.1 Small mammal abundance and diversity

We captured 544 individuals of 11 small mammal species over 7810 trap nights (Table 1). The deer mouse was by far the most frequently captured species, making up 78% of total captures. Trapping effort appeared to have been sufficient to indicate a species’ presence in each burn severity category (Figure S2). The recapture rate of marked individuals was similar among fire severity categories (Kruskal–Wallis *H*_2_ = 0.13, *p* = .94), suggesting that comparisons across categories were valid.

**TABLE 1:** Number of individual small mammals live‐captured across areas in different fire severity categories 3 years after the 2014 King Fire, California

Common name	Binomial	Unburned	Intermediate	High severity
Northern flying squirrel	*Glaucomys sabrinus*	2	0	0
Yellow‐pine chipmunk	*Neotoma amoenus*	0	3	1
Dusky footed woodrat	*Neotoma fuscipes*	4	1	0
Long‐eared chipmunk	*Neotamias quadrimaculatus*	4	11	2
Shadow chipmunk	*Neotamias senex*	0	8	4
California ground squirrel	*Otospermophilus beecheyi*	2	11	10
Brush mouse	*Peromyscus boylii*	12	2	4
North American deer mouse	*Peromyscus maniculatus*	103	106	217
Pinyon mouse	*Peromyscus truei*	1	0	1
Western harvest mouse	*Reithrodontomys megalotis*	2	0	0
Trowbridge's shrew	*Sorex trowbridgii*	29	5	0

**FIGURE 2:**
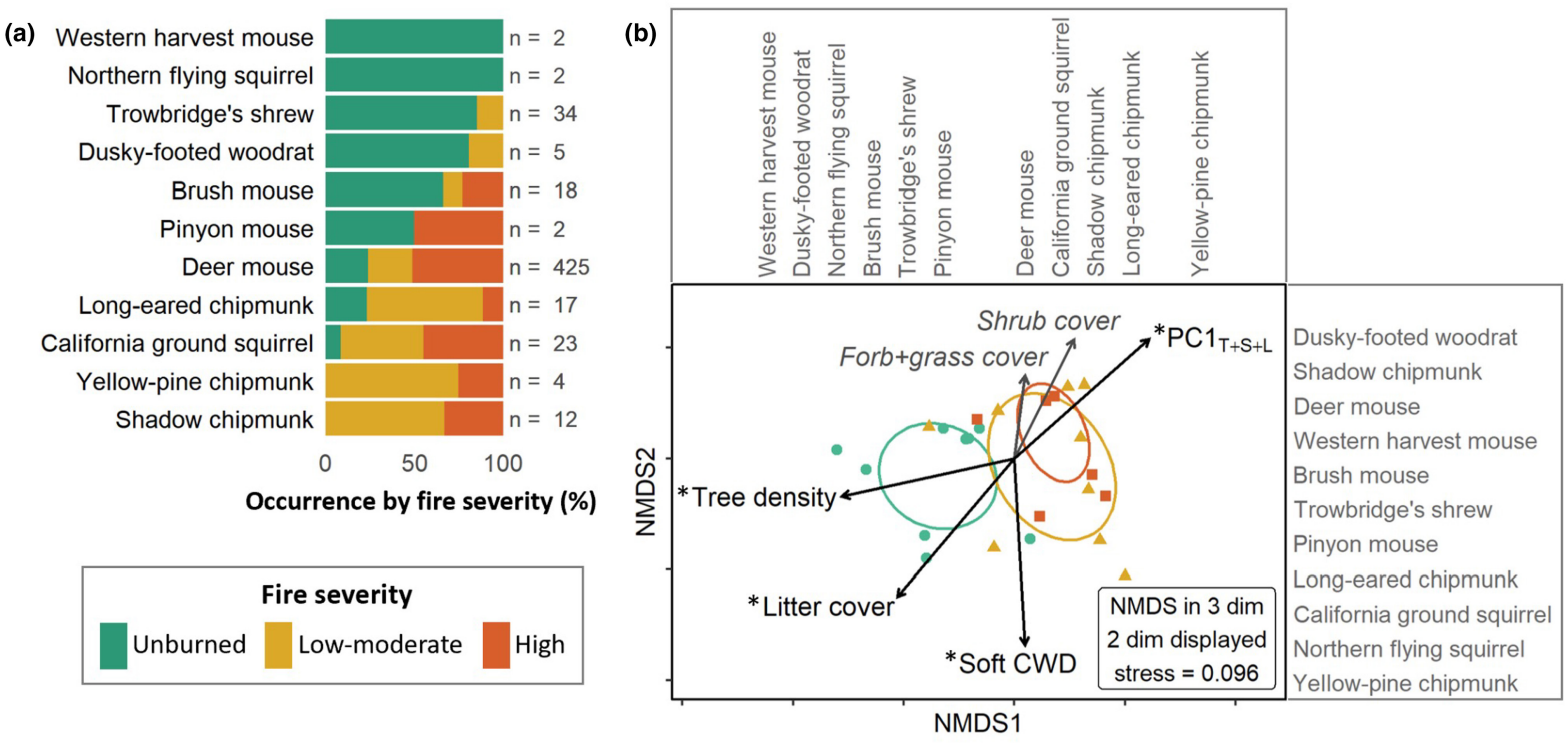
Small mammal community structure and habitat preferences across fire severity categories three years after the 2014 King Fire, California. (a) Bar plot showing the percentage of unique individuals trapped in each of the three fire severity categories for each of the 11 species captured, with the number of total captures denoted by *n*. (b) Nonmetric multidimensional scaling (NMDS) plot showing variation in the small mammal community structure across sites. Each point represents a site, with color‐coded ellipses encompassing ±1 standard deviation from the centroid for each category. Arrows represent vectors for vegetation variables, with significant correlations denoted by asterisks. The vegetation variables are soft coarse woody debris (CWD, m3/ha), shrub cover (% cover), forb/grass cover (% cover), litter cover (% cover), tree density (trees/hectare), and PC1T+S+L (representing the first axis of a principal components analysis of the three variables that changed with fire: live tree density, shrub cover, and litter cover). The 11 small mammal species are displayed along each NMDS axis according to their relative association with each axis

The relative abundances of different mammal species varied across fire severity categories (Figure 2a). Four species (deer mouse, long‐eared chipmunk, brush mouse, and California ground squirrel) were found in all three fire severity categories, with three (all except brush mouse) trapped more often within the two burned categories. Two species were found in both unburned and low/moderate‐severity sites (Trowbridge's shrew and dusky‐footed woodrat), but these species were more frequently caught in unburned habitats. Two rare species (northern flying squirrel and western harvest mouse) were caught exclusively at unburned sites, and two chipmunk species (yellow‐pine chipmunk and shadow chipmunk) were caught exclusively at burned sites. The pinyon mouse was a rare species that was caught once at an unburned site and once at a high‐severity site.

**FIGURE 3:**
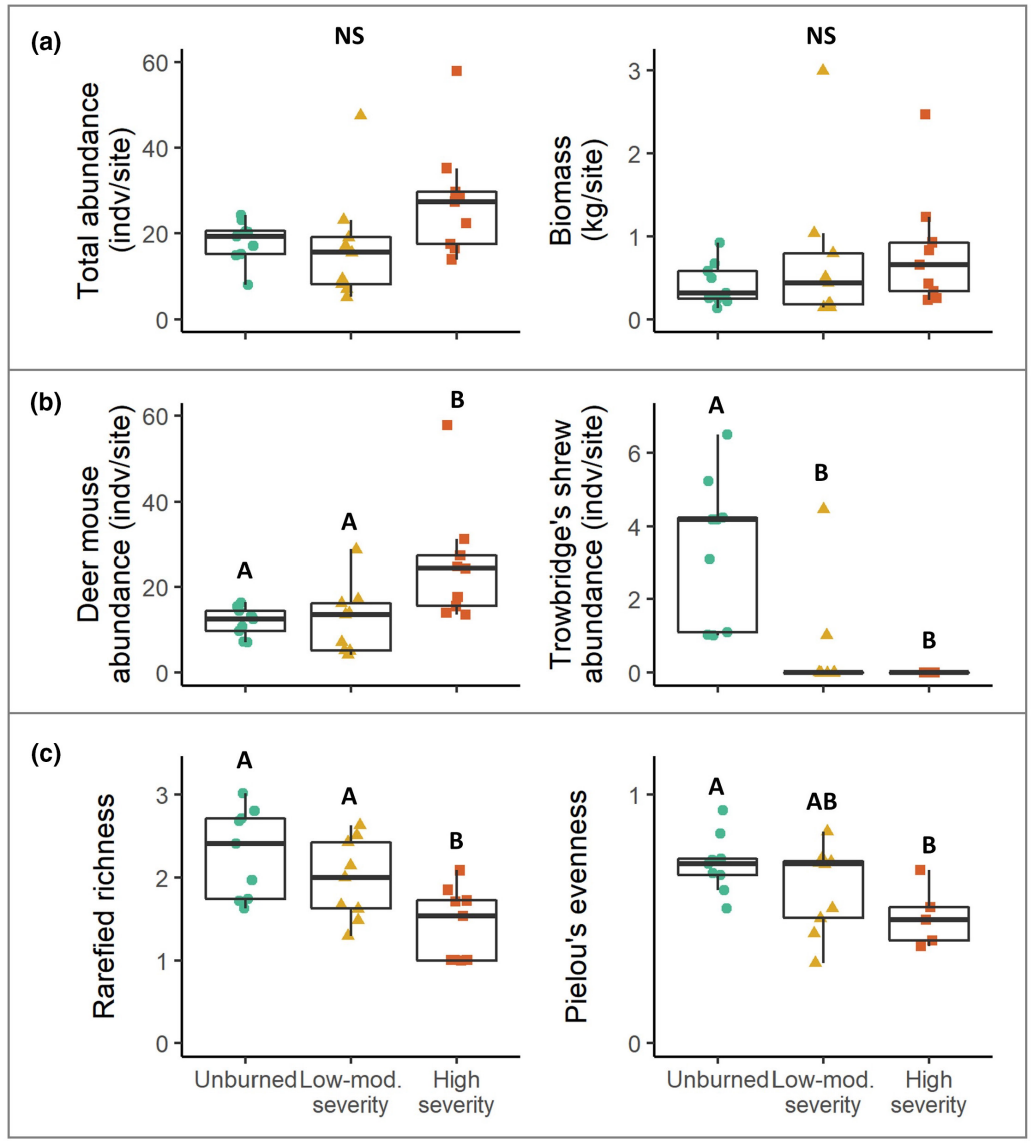
Abundance and diversity metrics across fire severity categories three years after the 2014 King Fire, California. Box plots show median and upper/lower quartiles. Categories in each plot with the same overlying letter are not significantly different. (a) Total small mammal abundance, calculated as the number of unique individuals captured over a 3‐day sampling period at each site, and total small mammal biomass did not differ among fire severity categories. (b) Individual species showed different responses to fire severity categories: deer mice were more abundant at high severity than other sites, whereas Trowbridge's shrews were more abundant at unburned than other sites. (c) Small mammal diversity, quantified as rarefied species richness (number of species per five individuals) and Pielou's index for species evenness, was lower at high severity sites than unburned sites

Total small mammal abundance did not differ among fire categories, although the median abundance was much higher at high‐severity sites (28 individuals per site) than at unburned or low/moderate‐severity sites (19 and 16 individuals per site, respectively) (Figure 3a, *H*_2_ = 5.44, *p* = .066, effect size = 0.14). Similarly, differences in total small mammal biomass among burn categories were not significant (Figure 3a, *H*_2_ = 1.45, *p* = .48, effect size = 0.023). Median deer mouse abundance, however, almost doubled from 13 individuals at unburned sites to 24 individuals at high‐severity sites (Figure 3b, *H*_2_ = 9.25, *p* < .01, effect size = 0.30, post hoc *p* < .01), although there were no differences in deer mouse abundance between unburned and low/moderate‐severity sites (post hoc *p* = .86). Shrew abundance showed the opposite relationship with fire severity, with median abundance decreasing from unburned sites (4 individuals) to low/moderate‐severity and high‐severity sites (0 individuals for both) (Figure 3b, *H*_2_ = 18.54, *p* < .001, effect size = 0.69, post hoc *p* < .01). Differences in shrew abundance between low/moderate‐severity and high‐severity sites were negligible (post hoc *p* = .19). No differences in the abundances of other species among burn severity categories were found.

Small mammal diversity as measured by rarified richness and evenness was lower at high‐severity than other sites. Rarefied species richness was lower at high‐severity sites compared with unburned and low/moderate‐severity sites (Figure 3c, ANOVA *F*_2,24_ = 7.19, *p* < .01, effect size = 0.77, post hoc *p* < .05), but rarified richness was similar between unburned and low/moderate‐severity sites (post hoc *p* = .36). Evenness also was lower in high‐severity sites compared with unburned sites (Figure 3c, ANOVA *F*_2,24_ = 3.65, *p* = .045, effect size = 0.60, post hoc *p* < .05), although evenness in low/moderate‐severity sites was statistically similar to that at both unburned and high‐severity sites (post hoc *p* = .31, .36, respectively).

### 3.2 Small mammal community structure

Small mammal community structure differed among fire severity categories (GLM_severity_, score_24,2_ = 31.73, *p* < .001). PCNM (accounting for spatial autocorrelation) was not a significant predictor in this model (score_23,1_ = 12.03, *p* = .13). Species‐specific responses showed that the deer mouse (score_24,2_ = 11.48, *p* < .01) and Trowbridge's shrew (score_24,2_ = 9.80, *p* < .01) were driving the community response to fire severity. Community structure varied among fire severity categories (Figure 2b, NMDS adonis *F*_2,24_ = 4.96, *R* ^2^ = 0.29, *p* = .001), with community structure at unburned sites being significantly different than at both low/moderate‐severity and high‐severity sites (post hoc *p* < .01).

### 3.3 Vegetation drivers

**FIGURE 4:**
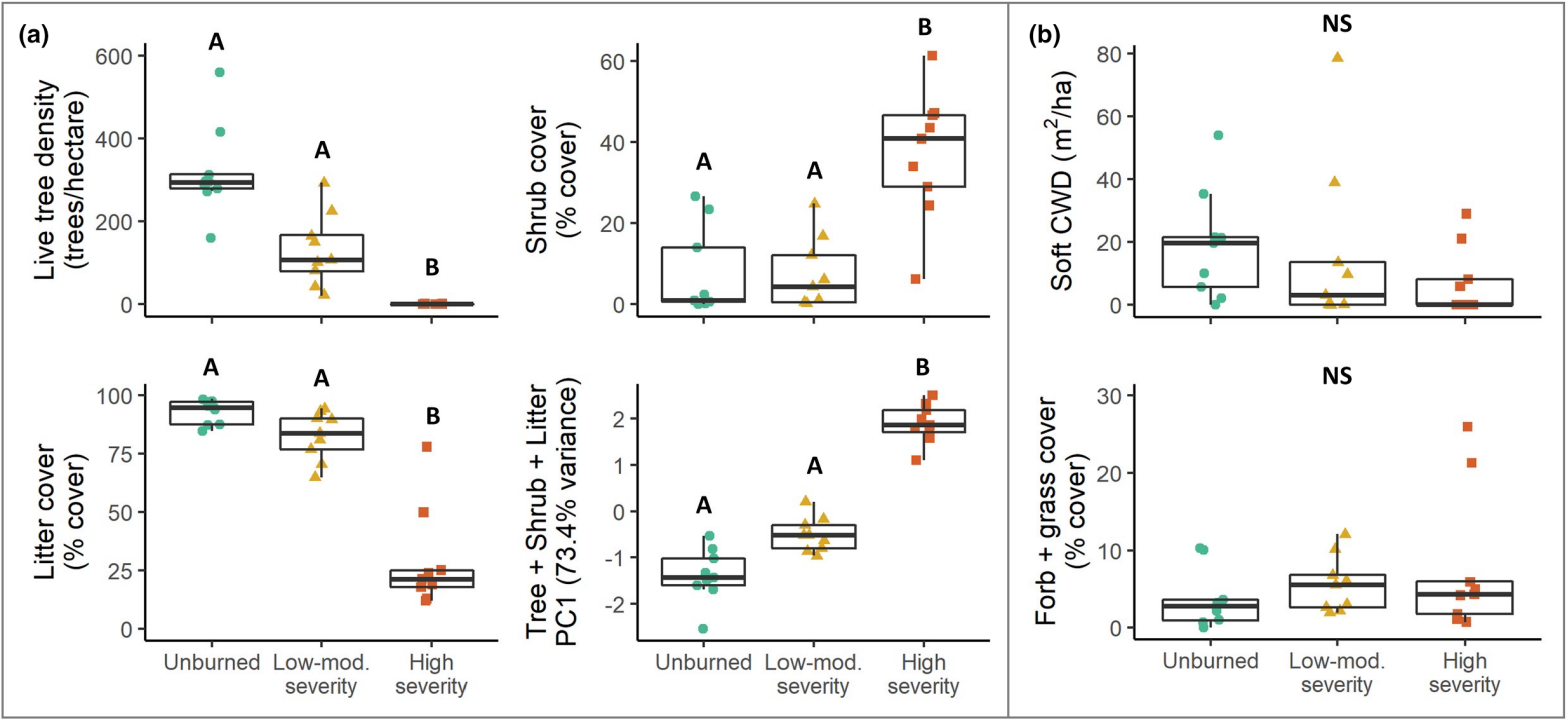
Differences in vegetation characteristics among fire severity categories three years after the 2014 King Fire, California. Box plots show median and upper/lower quartiles. Categories in each plot with the same overlying letter are not significantly different. (a) Live tree density and litter cover where lower at high‐severity sites than other sites, whereas shrub cover was higher at high‐severity sites. The first principal component (PC1T + S + L) of live tree density, shrub cover, and litter cover explained 73.4% of the variation in these three variables and was higher at high severity than other sites. (b) Volume of soft coarse woody debris and forb/grass cover was not different among fire severity categories

Out of the five vegetation variables that we predicted would affect small mammal community structure, three varied among the fire severity categories. The density of live trees varied most strongly among the fire severity categories, with high‐severity sites showing much lower density (Figure 4a, *H*_2_ = 22.26, *p* < .001, effect size = 0.84, post hoc *p* < .05). Percent litter cover also was lower at high‐severity than other sites (Figure 4a, *H*_2_ = 19.06, *p* < .001, effect size = 0.71, post hoc *p* < .05), and percent shrub cover was higher at high‐severity than other sites (Figure 4a, *H*_2_ = 14.14, *p* < .01, effect size = 0.51, post hoc *p* < .01). In addition, live tree density, litter cover, and shrub cover all appeared strongly aligned with the fire severity categories in the NMDS plot (Figure 4a). Unsurprisingly, these three variables were highly correlated (Spearman's correlation coefficients +0.66 to +0.74), so we collapsed them into the first principal component for use in GLM_veg_. The resulting PC1_T+S+L_ accounted for 73.4% of the variation in the three variables, and was higher at high‐severity than other sites (Figure 4a, Kruskal‐Wallis *H*_2_ = 21.60, *p* < .001, effect size = 0.82, post hoc *p* < .05).

The remaining two vegetation variables, soft CWD and forb/grass cover, did not vary among fire severity categories (Figure 4b, *H*_2_ = 1.90, *p* = .99 for CWD; *H*_2_ = 3.33, *p* = .95 for forb/grass cover). Furthermore, soft CWD and forb/grass cover were not strongly correlated with each other or with the other vegetation variables (Spearman's correlation coefficients <0.4).

The best model for predicting GLM_veg_ included PC1_T+S+L_ and soft CWD as predictors (AIC = 583.11, AIC weight = 0.94). No other model received substantial support and the final model displayed a good model fit according to Dunn–Smyth residuals and successfully predicted small mammal community structure (score_24,2_ = 37.57, *p* < .01). The strongest predictor of small mammal community structure (score_24,2_ = 27.40, *p* < .001) was PC1_T+S+L_with the volume of soft CWD also having a substantial, but much lower, predictive value (score_24,2_ = 16.72, *p* = .028).

### 3.4 Small mammal functional traits

**FIGURE 5:**
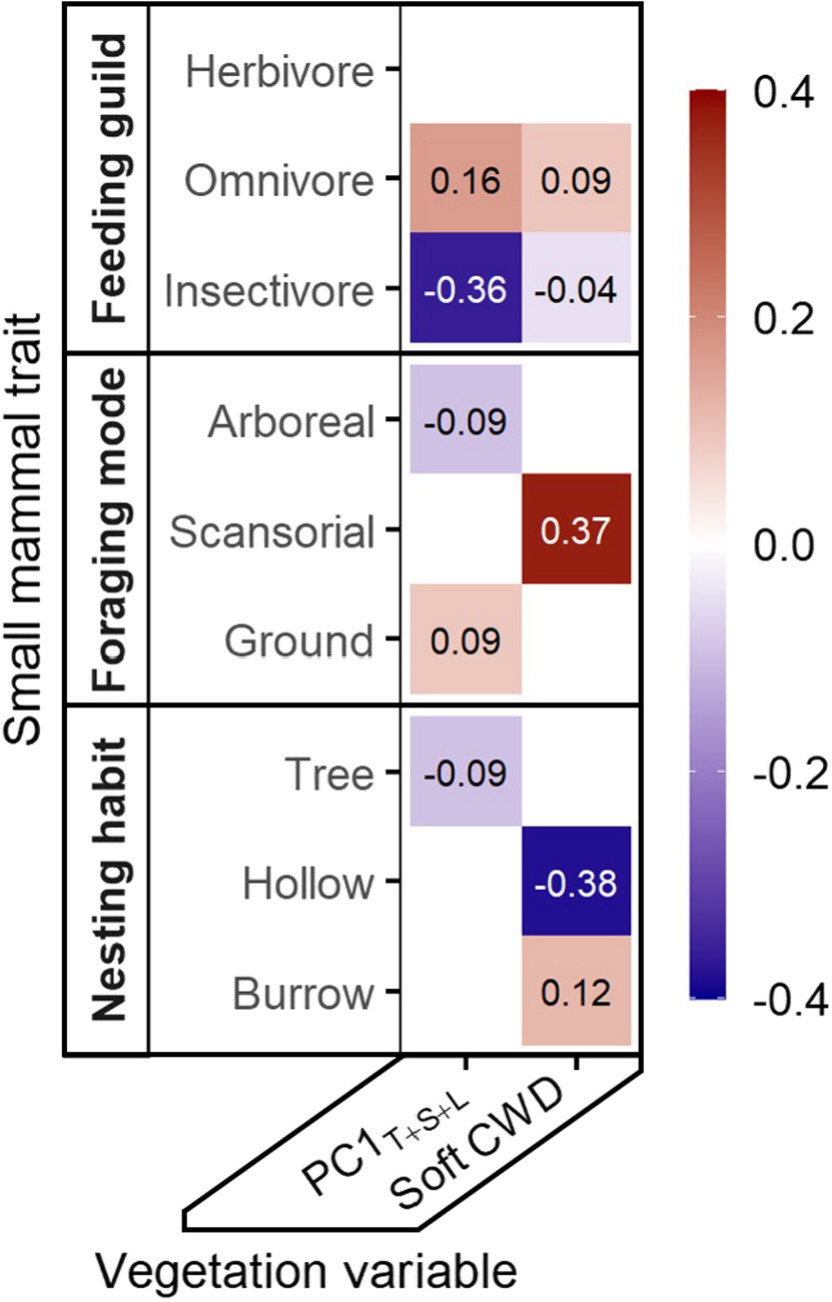
Interaction coefficients between small mammal traits and vegetation variables three years after the 2014 King Fire, California. The heat map shows standardized interaction coefficient estimates from a fourth‐corner model (GLMtrait) after variable selection using the LASSO penalty. Red (positive) and blue (negative) shading intensities represent the interaction strengths between small mammal traits and vegetation variables. Small mammal traits (feeding guild, foraging mode, and nesting habit) are categorical with levels designated on the *y*‐axis. The two vegetation variables are soft coarse woody debris (CWD m^3^/ha) and PC1T + S + L (representing the first axis of a principal component analysis for three vegetation variables that changed with fire: live tree density, shrub cover, and litter cover)

Small mammal community structure was successfully predicted by GLM_trait_ (Deviance_248,12_ = 60.89, *p* = .02), suggesting that relationships between vegetation variables and small mammal functional traits were important for determining community structure. Specifically, the interaction coefficients of GLM_trait_ showed several correlations between small mammal traits and vegetation variables (Figure 5). PC1_T+S+L_ was negatively correlated with insectivory (coefficient = −0.36), but positively correlated with omnivory (coefficient = 0.16). The volume of soft CWD was negatively correlated with nesting in hollows (coefficient = −0.38) but positively correlated with both scansorial foraging (coefficient = 0.37) and nesting in burrows (coefficient = 0.12).

## 4 DISCUSSION

With the risk of large high‐severity wildfires increasing across western North America, the 2014 King Fire provided an important opportunity to examine wildlife responses to “mega‐fires.” We examined the responses, and possible mechanisms for the responses, of small mammal communities to the King Fire after three years. We were able to predict small mammal community structure using the vegetation characteristics of sites varying in burn severity, and the traits of captured small mammal species, suggesting that post‐fire small mammal recovery is driven by small mammal resource use.

### 4.1 Fire effects on small mammals

Contrary to our expectation that small mammal abundance would increase at burned sites owing to the proliferation of deer mice, we did not find differences in overall mammalian abundance or biomass among sites in different fire severity categories. Much of the small mammal community response to high‐severity fire was driven by deer mice, which accounted for 78% of total captures and were more abundant at high‐severity than unburned sites. The pattern in deer mouse abundance matches that found in the literature, with abundance consistently increasing with fire severity (Fontaine & Kennedy, 2012; Krefting & Ahlgren, 1974; Zwolak, 2009). As generalist consumers, deer mice often invade disturbed habitats such as burned areas, clear‐cuts, mine waste piles, and the blast zones of volcanic eruptions (Andersen & MacMahon, 1985; Kirkland, 1976; Sullivan & Krebs, 1981). Nevertheless, total small mammal abundance was not different among burn categories, indicating that the abundance of other species obscured large differences in deer mouse abundance among burn categories.

In general, omnivores such as the deer mouse, long‐eared chipmunk, California ground squirrel, yellow‐pine chipmunk, and shadow chipmunk were captured more frequently at burned than unburned sites, although this was only significant for deer mice, likely due to low capture rates for other species. We found an association between omnivory and fire‐related changes in vegetation, indicating that generalists tend to fare better in post‐fire habitats.

In contrast, the abundance of Trowbridge's shrew was greater in unburned than both low/moderate‐ and high‐severity sites. Unlike deer mice, shrews tend to decrease in abundance after fire, especially high‐severity fire (Greenberg et al., 2007; Zwolak & Foresman, 2007), a disturbance that removes leaf litter, which hosts their invertebrate prey (Greenberg et al., 2007). Consistent with this pattern, we found a large decrease in litter cover at high‐severity than other sites; however, litter cover was similar between unburned and low/moderate‐severity sites. Given the mismatch between high litter cover and low shrew abundance in low/moderate‐severity sites, another habitat variable is likely driving shrew abundance in these habitats. A plausible driver is soft CWD, which serves as another habitat for invertebrates (Jia‐bing et al., 2005). However, our data do not show any strong differences in soft CWD cover among fire severity categories, and our fourth‐corner analysis showed a slight negative correlation between insectivory and soft CWD. As a consequence, the reasons for low shrew densities at burned sites remain unclear.

Several uncommonly captured species (e.g., the dusky‐footed woodrat and the northern flying squirrel) also were exclusively or disproportionately captured in unburned habitats. This is likely a real effect given that these species use woodpile dens and depend on high tree density, respectively. Our sampling design, however, was inadequate for effectively censusing these species and, therefore, we caution against interpreting a lack of significant response in these species as evidence for a lack of effect.

Small mammal richness and evenness were lower in high severity compared with unburned sites, consistent with our initial hypotheses and the findings of other studies (Fisher & Wilkinson, 2005; Zwolak & Foresman, 2007). We did not find differences in richness and evenness between low/moderate‐severity and unburned sites, however. In addition, we found that there were no differences between low/moderate‐severity and unburned sites in vegetation variables partly due to substantial variation among sites within the same fire severity category. Some fire effects on vegetation at low/moderate‐severity sites also may have dissipated in the three years between the fire and our sampling, highlighting a limitation of this study, which was done at a single point in time. The effects of high‐severity fire are likely more long‐lasting than the effects of low/moderate‐severity fire with previous studies showing that small mammal responses to low‐severity fire dissipate within <2 years (Horncastle et al., 2019). Our analyses also were limited by our abundance metric, the minimum number of animals known to be alive (MNKA), considering the low numbers of many species collected. Although this method does not account for differences in the detectability of different small mammal species, which may bias evenness metrics, our evenness results are consistent with the rarified richness responses (not limited by MNKA) so we believe that this result is robust. Interestingly, overall small mammal community structure was similar between low/moderate‐severity and high‐severity sites, which differed from unburned sites. This pattern may have been driven largely by the Trowbridge's shrew, the second most frequently trapped mammal, which was virtually absent at all burned sites.

### 4.2 Mechanisms

In general, small mammal community structure across burn categories was highly correlated with vegetation characteristics (Schmid‐Holmes & Drickamer, 2001). Understory vegetation cover serves as protection from predators (Powell & Banks, 2004; Torre & Díaz, 2004), and provides key food resources such as seeds, fruits, and vegetative matter, which are especially important to rodents (Reid, 2006; Whitaker, 1996). In mixed conifer forests, live trees can be a major seed food source, even for ground‐dwelling species, and also may help animals escape terrestrial predators (Lobo, 2014). Semi‐arboreal mammals such as woodrats and flying squirrels are even more dependent on live trees for nesting and food storage (Innes et al., 2007; Smith, 2007). Well‐decayed coarse woody debris hosts a variety of mammal food items such as fungi and insects (Jia‐bing et al., 2005), and provides cover from predators and nesting space for small mammals (Fauteux et al., 2012; McComb, 2003). For shrews, leaf litter also provides cover from predators and a habitat for invertebrates (Greenberg et al., 2007; MacCracken et al., 1985).

Because post‐fire changes in live tree density, shrub cover, and litter cover were highly correlated at our sites, we could not reliably tease apart their effects on small mammals. In combination, however, these three variables appeared to be strong drivers of small mammal community structure. Previous studies have found that post‐fire shrub cover is often associated with shifts in small mammal community structure, in part because it provides protection from predators (Borchert et al., 2014; Converse, Block, et al., 2006). Other studies, however, have found similar changes in small mammal communities only 1‐year post‐fire, when shrubs have not yet been established or grown (Zwolak & Foresman, 2007). Similarly, our analyses showed a negative correlation between arboreal foraging and tree‐nesting, and fire‐induced tree loss. Although some of these tree‐associated species were captured in low numbers, these results are consistent with other findings (e.g., flying squirrels avoiding disturbed forest sites, (Sollmann et al., 2016)).

Soft CWD also predicted small mammal community structures at our sites, although we did not find differences in CWD levels among fire severity categories. In contrast, previous studies have found that forest fires usually cause decreases in coarse woody debris (Converse, Block, et al., 2006; Knapp et al., 2005). Because the volume of CWD can quickly increase after fire as burned trees fall, this discrepancy may be due to the timing of our study, which took place three years after fire (Grayson et al., 2019).

Similarly, we did not observe any differences in forb/grass cover among fire severity categories. Differences in forb and grass cover among burn severity categories likely would be more apparent in the first growing seasons after the fire (Converse, Block, et al., 2006) when herbaceous cover would benefit from reduced overstory competition for light. This temporary stimulation of grass and forbs quickly dissipates as shrubs establish, grow, and shade out forbs and grasses. These results underscore the importance of considering the relative time scales of different vegetation recovery processes when analyzing habitat (and wildlife) recovery post‐fire and highlight the need for additional studies of this type at various times after fire.

## 5 CONCLUSIONS

Three vegetation variables (density of live trees, shrub cover, and litter cover) that varied with fire severity were significant predictors of small mammal community structure and the structure of the post‐fire small mammal community was associated with habitat‐mammal resource use interactions.

These vegetation variables represent resources used as small mammal food, as a habitat for nesting, and as refuges from predators, but our analysis does not allow us to disentangle the reasons for small mammal responses to fire. It is also possible that small mammals are driving vegetation community structure via their trophic and dispersal roles (e.g., through selective seed predation or dispersal), and responding to vegetation conditions. Although we emphasize post‐fire small mammal responses as mediated through mammal resource use, additional experimental work will be needed to disentangle bottom‐up and top‐down causal pathways. Finally, we also stress the need for research in additional systems, and across multiple and longer time scales, to examine the generality of our results.

Mechanistic understanding of ecological responses to wildfire severity is critical for the conservation and management of fire‐prone systems (Freeman et al., 2017), especially given the increasing frequency of high‐severity fires across western North America (Schoennagel et al., 2017; Yue et al., 2013) and at middle to high latitudes globally (Moritz et al., 2012). Our results show substantial differences between the effects of low/moderate‐severity and high‐severity fires, both in habitat structure and small mammal community responses, suggesting that post‐fire management prescriptions promote small mammal diversity need to consider fire severity patterns.

## AUTHOR CONTRIBUTIONS

**Kathryn Culhane:** Data curation (lead); Formal analysis (lead); Investigation (equal); Visualization (lead); Writing – original draft (lead); Writing – review & editing (equal). **Rahel Sollmann:** Funding acquisition (lead); Investigation (equal); Project administration (lead); Supervision (supporting); Writing – review & editing (supporting). **Angela M. White:** Funding acquisition (supporting); Investigation (supporting); Project administration (equal); Writing – review & editing (supporting). **Gina L. Tarbill:** Investigation (supporting); Project administration (supporting); Writing – review & editing (supporting). **Scott D. Cooper:** Formal analysis (supporting); Supervision (supporting); Writing – review & editing (supporting). **Hillary S. Young:** Conceptualization (equal); Formal analysis (supporting); Funding acquisition (equal); Methodology (supporting); Project administration (supporting); Supervision (lead); Visualization (supporting); Writing – original draft (supporting); Writing – review & editing (equal).

## Supporting information

Supplementary Materials

## Data Availability

Data Accessiblity: All mammal trapping and vegetatiom data uploaded online at https://doi.org/10.25349/D9HW4P.
