# Supplementary material for: Small mammal responses to fire severity mediated by vegetation characteristics and species traits
Source: Ecol Evol. 2022 May 19;12(5):e8918. doi: 10.1002/ece3.8918 (PMC9120878; doi:10.1002/ece3.8918)
Supplement: Supplementary file 1 — Supplementary Materials [file ECE3-12-e8918-s001.doc]

## APPENDIX S1

## Site classification

We established the similarity of vegetation at our sites before the King Fire and compared pre-fire to post-fire conditions using spatial data products from the Landscape Fire and Resource Management Planning Tools program (LANDFIRE) developed in 2012 (LF 1.3.0) and 2014 (LF 1.4.0) (Rollins & Frame, 2006). In particular, we used two metrics from LANDFIRE: Existing Vegetation Cover (EVC), which is a measure of the percent cover of the dominant life forms (tree, shrub, or herb) within 30-m cells, and Existing Vegetation Type (EVT), which classifies the dominant terrestrial ecological system within 30-m cells as described by NatureServe (Comer et al., 2003). To compare potential wildlife habitat across sites, we converted the EVT classes into California Wildlife Habitat Relationships (CWHR) habitat classes (Comer et al., 2003). For each site, we counted the number of cells within each vegetation cover category (for EVC) or vegetation type (for CWHR, converted from EVT). We included all cells within each mammal trapping site (90 × 90 m) plus a buffer of 225 m (the diameter of the largest home range of captured species according to values from the PanTHERIA database, (K. E. Jones et al., 2009)), excluding cells from vegetation types that comprised <1% of the area sampled for each time period.

To quantitatively compare EVC among the three fire severity categories for both pre-fire and post-fire periods, we used ordinal ranks for the percent cover of trees based on the categories provided by LANDFIRE, with cell values representing dominance by shrubs and herbs lumped into the lowest rank. We compared ranked EVC sums using two-tailed Kruskal-Wallis tests with a Bonferroni correction. Effect size was calculated as eta squared using the H-statistic and post hoc pairwise comparisons among burn severity categories were conducted using Bonferroni-corrected Dunn’s tests. To test differences between pre-fire and post-fire EVC for each of the three fire severity categories, we used three Bonferroni-corrected Wilcoxon ranked sum tests. Wilcoxon effect size was calculated as the r value.

Given the sampling design and the difficulty of using any one metric to describe fire severity, all analyses in this paper were conducted using categorical bins of fire severity (high severity, low/moderate severity, and unburned) rather than a quantitative site-specific severity score. To validate that these bins distinguished different fire severity categories, we used both remote sensing and field data. We used remote sensing data from Monitoring Trends in Burn Severity (MTBS) maps, which classify burn severity using Landsat reflectance imagery of pre-fire and post-fire conditions at 30-m resolution (Eidenshink et al., 2007). MTBS classifies severity into five possible scores (0 = outside the fire boundary; 1 = unburned-low severity burn within the fire perimeter; 2 = low severity; 3 = moderate severity; 4 = high severity). For each treatment, we determined the mean MTBS pixel value for each mammal trapping site and its home range buffer.

In the field, we also tested for differences in fire-related tree mortality among severity categories based on surveys of trees along two 50-m vegetation transects at each site (simultaneous to mammal trapping work). For all trees with boles within a 15-m wide band (750 m^2^ per transect) and with a diameter at breast height (DBH) greater than 15 cm, we recorded if the tree was alive or dead. We calculated percent tree mortality as the number of dead trees divided by the total. We compared percent tree mortality and average severity pixel value per site among the three fire severity categories using Kruskal-Wallis tests followed by Bonferroni-corrected Dunn’s tests.

*Results from site classifications*

Before the King Fire in 2014, vegetation at the 27 sites was similar across the three fire severity categories, with habitats consisting predominantly of Sierran mixed conifer forest or other conifer forest types in 2012 (**Fig. S1b**). Although pre-fire tree cover was highly variable across sites (range 20–80%), differences were minimal among the three fire severity categories, with a small effect size (**Fig. S1a**, Kruskal-Wallis H_2_ = 12.3, p < 0.01, effect size = 0.006).

Three years after the 2014 King Fire, vegetation had shifted from mixed conifer forest to montane chaparral, with the most drastic shifts occurring at the high-severity sites (**Fig. S1b**). We observed a large difference between pre-fire and post-fire percent cover of trees in high-severity sites (Wilcoxon W = 3420, p < 0.001, effect size = 0.94). In comparison, pre-fire and post-fire tree cover was only moderately different at low/moderate-severity sites (Wilcoxon W = 172929, p < 0.001, effect size = 0.33), and indistinguishable at unburned sites (Wilcoxon W = 149933, p = 0.57, effect size = 0.039).

The three fire severity categories also differed in vegetation cover after the King Fire. Immediately post-fire in 2014, the percent cover of trees among the three fire severity categories differed greatly (**Fig. S1a**, Kruskal-Wallis H_2_ = 1535, p < 0.001, effect size = 0.66, all post hoc p < 0.001). In addition, sites in the three fire severity categories showed strong differences in average fire severity, as measured from MTBS pixels (**Fig. 1c**, Kruskal-Wallis H_2_ = 23.4, p < 0.001, effect size = 0.89, all post hoc p < 0.05). Three years after the fire, these differences were still distinct, with different tree mortality levels among treatments according to 2017 field data (**Fig. 1c**, Kruskal-Wallis H_2_ = 25.0, p < 0.001, effect size = 0.96, all post hoc p < 0.05).

**Table S1. Trait values and references for each of the 11 small mammal species collected in this study. Each species was assigned to one of three traits in each trait category (feeding mode, foraging area, and nesting habitat)**. Herbivores are defined as having 80% or more of their diet from plants, insectivores as having 80% or more of their diet from insects, and omnivores having a mixed value. Full references are provided below the table.

| **Species** | **Traits** | **Reference(s)** |
| --- | --- | --- |
| *Glaucomys sabrinus*  (northern flying squirrel) | Omnivore | Reid, 2006; Wells-Gosling & Heaney, 1984; Whitaker, 1996 |
|  | Arboreal |  |
|  | Tree |  |
| *Neotamias amoenus*  (yellow-pine chipmunk) | Omnivore | Reid, 2006; Sutton, 1992; Whitaker, 1996 |
|  | Scansorial |  |
|  | Burrow |  |
| *Neotamias quadrimaculatus*  (long-eared chipmunk) | Omnivore | Clawson et al., 1994; Reid, 2006; Whitaker, 1996 |
|  | Scansorial |  |
|  | Tree |  |
| *Neotamias senex*  (shadow chipmunk) | Omnivore | Gannon & Forbes, 1995; Reid, 2006; Whitaker, 1996 |
|  | Ground |  |
|  | Tree |  |
| *Neotoma fuscipes*  (dusky-footed woodrat) | Herbivore | Carraway & Verts, 1991; Reid, 2006; Whitaker, 1996 |
|  | Arboreal |  |
|  | Tree |  |
| *Otospermophilus beecheyi*  (California ground squirrel) | Omnivore | Reid, 2006; Smith et al., 2016; Whitaker, 1996 |
|  | Ground |  |
|  | Burrow |  |
| *Peromyscus boylii*  (brush mouse) | Omnivore | Kalcounis-Rueppell & Spoon, 2009; Reid, 2006; Whitaker, 1996 |
|  | Scansorial |  |
|  | Hollow |  |
| *Peromyscus maniculatus*  (deer mouse) | Omnivore | Reid, 2006; Whitaker, 1996 |
|  | Scansorial |  |
|  | Hollow |  |
| *Peromyscus truei*  (pinyon mouse) | Herbivore | Hoffmeister, 1981; Reid, 2006; Whitaker, 1996 |
|  | Scansorial |  |
|  | Hollow |  |
| *Reithrodontomys megalotis*  (western harvest mouse) | Herbivore | Reid, 2006; Webster & Jones, 1982; Whitaker, 1996 |
|  | Scansorial |  |
|  | Hollow |  |
| *Sorex trowbridgii*  (Trowbridge’s shrew) | Insectivore | George, 1989; Reid, 2006; Whitaker, 1996 |
|  | Ground |  |
|  | Burrow |  |

Carraway, L. N., & Verts, B. J. (1991). Neotoma fuscipes. *Mammalian Species*, *386*, 1–10. https://doi.org/10.2307/3504130

Clawson, R. G., Clawson, J. A., & Best, T. L. (1994). Tamias quadrimaculatus. *Mammalian Species*, *469*, 1–6.

Elbroch, M., & Rinehart, K. (2011). *Behavior of North American mammals*. Houghton Mifflin Harcourt.

Flynn, D. F. B., Gogol-Prokurat, M., Nogeire, T., Molinari, N., Richers, B. T., Lin, B. B., Simpson, N., Mayfield, M. M., & DeClerck, F. (2009). Loss of functional diversity under land use intensification across multiple taxa. *Ecology Letters*, *12*(1), 22–33. https://doi.org/10.1111/j.1461-0248.2008.01255.x

Gannon, W. L., & Forbes, R. B. (1995). Tamias senex. *Mammalian Species*, *502*, 1–6.

George, S. B. (1989). Sorex trowbridgii. *Mammalian Species*, *337*, 1–5. https://doi.org/10.2307/3504159

Hoffmeister, D. F. (1981). Peromyscus truei. *Mammalian Species*, *161*, 1–5. https://doi.org/10.2307/3503851

Kalcounis-Rueppell, M. C., & Spoon, T. R. (2009). Peromyscus boylii (Rodentia: Cricetidae). *Mammalian Species*, *838*, 1–14. https://doi.org/10.1644/838.1

Reid, F. (2006). *Peterson field guide to mammals of North America* (4th ed.). Houghton Mifflin Harcourt.

Smith, J. E., Long, D. J., Russell, I. D., Newcomb, K. L., & Muñoz, V. D. (2016). Otospermophilus beecheyi. *Mammalian Species*, *48*(939), 91–108. https://doi.org/10.1093/mspecies/sew010

Sutton, D. A. (1992). Tamias amoenus. *Mammalian Species*, *390*, 1–8.

Webster, Wm. D., & Jones, J. K. (1982). Reithrodontomys megalotis. *Mammalian Species*, *167*, 1–5. https://doi.org/10.2307/3504020

Wells-Gosling, N., & Heaney, L. R. (1984). Glaucomys sabrinus. *Mammalian Species*, *229*, 1–8. https://doi.org/10.2307/3503926

Whitaker, J. O. (1996). *National Audubon Society field guide to North American mammals* (2nd ed.). Alfred A. Knopf Inc.

Wilman, H. A. C. (2011). *The energetic niche of species: Integrating single-species and ecosystem perspectives on species’ role in communities*. UC San Diego.

**Supplementary Figure 1**


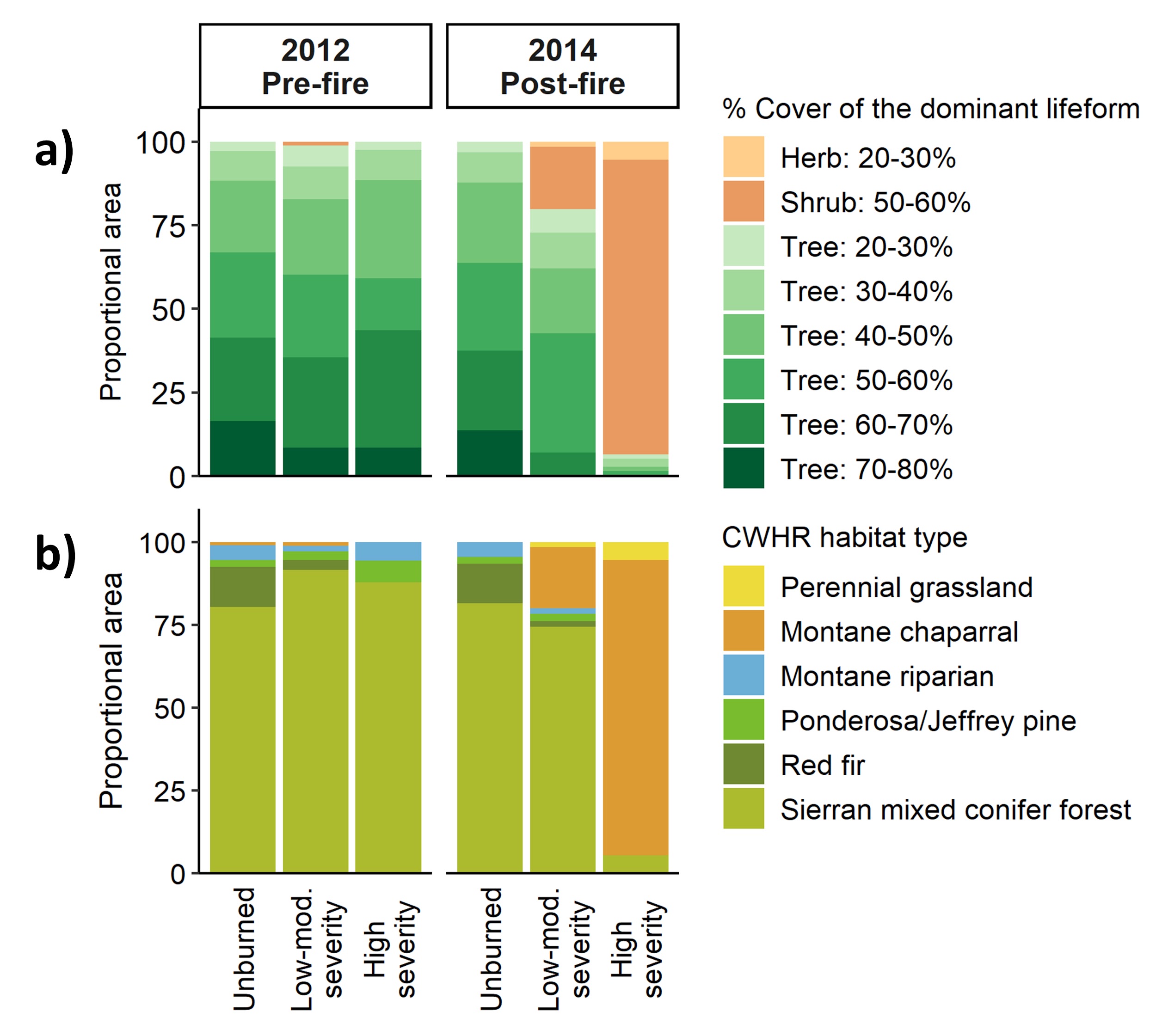


**Fig S1. Vegetation characteristics were similar across fire severity categories before (2012) but differed greatly after (2014) the King fire, California.** For each fire severity category, stacked bars show the percentage of 30-m cells across nine sites by **(a)** percent cover classes of the dominant life form and **(b)** habitat type. Details in Supp Material 1.

**Supplementary Figure 2**


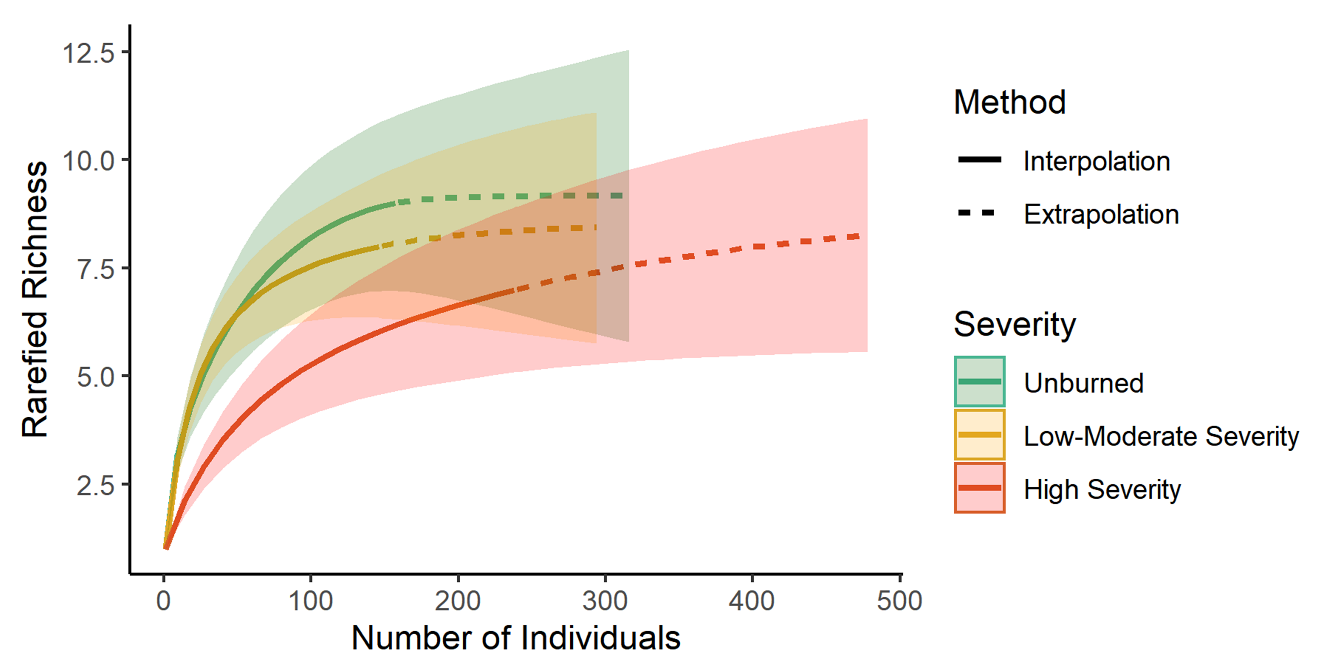


**Fig. S2.** Rarefaction curves for small mammal richness for the three fire severity categories. The rarefied curves indicate the number of species detected per trapped individuals, with observations pooled by treatment. Shaded areas represent unconstrained 95% confidence intervals.
